# O-Net: A Novel Framework With Deep Fusion of CNN and Transformer for Simultaneous Segmentation and Classification

**DOI:** 10.3389/fnins.2022.876065

**Published:** 2022-06-02

**Authors:** Tao Wang, Junlin Lan, Zixin Han, Ziwei Hu, Yuxiu Huang, Yanglin Deng, Hejun Zhang, Jianchao Wang, Musheng Chen, Haiyan Jiang, Ren-Guey Lee, Qinquan Gao, Ming Du, Tong Tong, Gang Chen

**Affiliations:** ^1^College of Physics and Information Engineering, Fuzhou University, Fuzhou, China; ^2^Fujian Key Lab of Medical Instrumentation and Pharmaceutical Technology, Fuzhou University, Fuzhou, China; ^3^Department of Pathology, Fujian Cancer Hospital, Fujian Medical University, Fuzhou, China; ^4^College of Electrical Engineering and Automation, Fuzhou University, Fuzhou, China; ^5^Department of Electronic Engineering, National Taipei University of Technology, Taipei, Taiwan; ^6^Imperial Vision Technology, Fuzhou, China; ^7^Fujian Provincial Key Laboratory of Translational Cancer Medicin, Fuzhou, China

**Keywords:** CNN, transformer, medical image segmentation, deep learning, classification

## Abstract

The application of deep learning in the medical field has continuously made huge breakthroughs in recent years. Based on convolutional neural network (CNN), the U-Net framework has become the benchmark of the medical image segmentation task. However, this framework cannot fully learn global information and remote semantic information. The transformer structure has been demonstrated to capture global information relatively better than the U-Net, but the ability to learn local information is not as good as CNN. Therefore, we propose a novel network referred to as the O-Net, which combines the advantages of CNN and transformer to fully use both the global and the local information for improving medical image segmentation and classification. In the encoder part of our proposed O-Net framework, we combine the CNN and the Swin Transformer to acquire both global and local contextual features. In the decoder part, the results of the Swin Transformer and the CNN blocks are fused to get the final results. We have evaluated the proposed network on the synapse multi-organ CT dataset and the ISIC 2017 challenge dataset for the segmentation task. The classification network is simultaneously trained by using the encoder weights of the segmentation network. The experimental results show that our proposed O-Net achieves superior segmentation performance than state-of-the-art approaches, and the segmentation results are beneficial for improving the accuracy of the classification task. The codes and models of this study are available at https://github.com/ortonwang/O-Net.

## 1. Introduction

Image enhancement has been extensively performed on medical images based on morphology, such as clustering (Vasuda and Satheesh, 1713), edge detection (Patil and Deore, 2013), and threshold segmentation (Wang et al., 2015) to assist doctors in diagnosis in the early days. With the development of artificial intelligence, deep learning technology has been widely used in medical image processing and analysis in recent years, and the accuracy of segmentation and classification on medical images is of great significance to the diagnosis of diseases today. In clinical practice, accurate image segmentation can provide clinicians with quantitative information, which can help clinicians make diagnostic decisions more precisely and efficiently (Liang et al., 2020). In addition, the additional information provided by computing methods is subjective and can avoid the objective bias by humans.

Nowadays, Convolutional Neural Network (CNN), especially Full Convolutional Network (FCN) is an effective segmentation method (Wang et al., 2021b) and it has been widely used in dense classification tasks such as semantic segmentation (Ji et al., 2020). Among different CNN networks, U-Net (Ronneberger et al., 2015) is a deep learning network with encoder and decoder structures, which has been widely used in medical image segmentation. In recent years, it has been widely used in medical image segmentation tasks due to its strong generalization. U-Net and its variants UNet++ (Zhou et al., 2018), UNet 3+ (Huang et al., 2020), CE-Net (Gu et al., 2019) have shown excellent performance in tasks, such as lesion segmentation, heart segmentation, and other organ segmentation. Based on the strong ability of learning and discriminating features, Res-UNet (Xiao et al., 2018) improves the performance of the network by introducing a residual network into the encoder part of U-Net. EfficientNet (Tan and Le, 2019) proposed a new scaling method that uniformly all dimensions of the depth, width, and resolution of the network through simple but efficient composite coefficients, which not only reduces a certain amount of calculation, but also improves the segmentation performance. Many experimental results have shown that the use of EfficientNet as an encoder can often further improve the performance of the network without increasing the amount of calculation.

However, these networks are faced with the common problem of CNN: it is difficult for CNN-based methods to learn the global and remote semantic information interaction (Chen et al., 2021) clearly. This is due to the fact that CNN extracts features with a convolutional process. Some studies tried to use image feature pyramid (Lin et al., 2017), atrous convolution layers (Chen et al., 2017, 2018; Gu et al., 2019), and self-attention mechanisms (Wang et al., 2018; Schlemper et al., 2019) to solve this problem. However, the global and remote semantic information is not fully learnt using these strategies. Inspired by the great success of transformer (Vaswani et al., 2017) in the field of natural language processing (NLP), researchers have tried to introduce transformer to make up for the shortcomings of CNN in global and remote information interaction. A transformer is an attention-based model and self-attention mechanism (SA) is a key component of transformer. It can model the correlation of all input tags which makes room for the transformer to deal with long-range dependencies. In Dosovitskiy et al. (2020), vision transformer (ViT) was applied to perform image recognition tasks and achieved relatively good results. After that, a novel framework called Swin Transformer (Liu et al., 2021) was proposed and significantly improved the performance of ViT in different tasks, such as image classification (Liu et al., 2021), object detection (Xu et al., 2021), and semantic segmentation (Xie et al., 2021). Based on the Swin Transformer, Cao et al. (2021) proposed Swin-Unet, which combined the U-Net structure and Swin Transformer for medical image segmentation, the encoding part and the decoding part in Swin-Unet were both performed using Swin Transformer. With the proposal of these methods, the accuracy of segmentation tasks is further improved. However, the input in transformer is formed as one-dimensional sequence. The transformer networks focus on learning the global contextual information, but may lose some local details. Therefore, it is beneficial to combine the global information learnt by transformer and the local information by CNN to enrich the learnt features.

Based on the advantages of CNN and transformer, we propose an O-Net framework to combine the CNN and the transformer to learn both global and local contextual features. We combine the CNN and Swin Transformer as encoder first and send them into a CNN-based decoder and a Swin Transformer-based decoder, respectively. The results of two decoders are fused to get the final result. This network combines the advantages of CNN and transformer and may improve the performance of medical image segmentation. Our experimental results have shown that the performance of the network can be significantly improved by combining CNN and transformer. In addition, a classification task is simultaneously performed based on the O-Net. Experiments show that the segmentation results are beneficial for improving the accuracy of the classification task. Experiments on the synapse multi-organ segmentation dataset and the ISIC2017 skin lesion challenge dataset have demonstrated the superiority of our method compared to other state-of-the-art segmentation methods. In addition, based on the segmentation network, the performance of the classification network has also been greatly improved.

## 2. Related Works

**CNN-based methods:** CNN is a kind of feedforward neural network that includes convolution calculations and has a deep structure. It is one of the representative algorithms of deep learning. Lenet[18] first defined the CNN network structure in 1998, and it was not until the publication of AlexNet (Krizhevsky et al., 2012) in 2012 that CNN has gradually become mainstream. Since then, lots of efficient and deep convolutional neural networks have been proposed. For example, VGG (Simonyan and Zisserman, 2014), ResNet (He et al., 2016), DenseNet (Huang et al., 2017), GoogleNet (Szegedy et al., 2015), HRNet (Sun et al., 2019), Inception v3 (Szegedy et al., 2016), and EfficientNet (Tan and Le, 2019). These networks perform well in various applications. In addition to these network innovations, new convolutional layers such as deformable convolution (Dai et al., 2017; Zhu et al., 2019) and depth-wise convolution (Xie et al., 2017) were proposed for different tasks. With the development of CNN, U-Net was proposed and widely used in segmentation tasks because of its simple structure, good effects, and strong generalization. After that, various U-shape network based U-Net have been proposed such as U-SegNet (Kumar et al., 2018), Res-UNet (Xiao et al., 2018), Dense-UNet (Li et al., 2018), U-Net++ (Zhou et al., 2018), U-2-Net (Qin et al., 2020), and UNet3+ (Huang et al., 2020) CE-Net (Gu et al., 2019). Gehlot et al. (2020) proposed an Encoder-Decoder based CNN with Nested-Feature Concatenation (EDNFC-Net) for automatic segmentation. Some networks introduce novel structures in the encoder part while others in the decoder part. Because of the strong generalization of the network, the U-shaped architecture network has also been extended to 3D medical image segmentation, such as 3D-UNet (Çiçek et al., 2016) and V-Net (Milletari et al., 2016). Moreover, Gehlot et al. proposed AION (Gehlot and Gupta, 2021), an architecture with two coupled networks and classification heads which is applicable for stain normalization, classification, and segmentation tasks.

**Transformers:** Transformer was first proposed for machine translation and achieved the best performance in many NLP tasks. To combine computer vision (CV) and natural language processing (NLP) domain knowledge, researchers developed Vision Transformer (ViT) (Dosovitskiy et al., 2020) by directly applying transformers with global self-focus to full-size images. The ViT model achieved both high efficiency and accuracy in image recognition tasks. Based on ViT, Chen et al. (2021) proposed the first transformer-based medical image segmentation framework TransUNet which further improved the accuracy of image segmentation tasks. However, ViT needs to be pre-trained on its large datasets to achieve good performance. To solve this problem, some training schemes were designed in Deit (Touvron et al., 2021) so that the algorithm can perform well on smaller data sets. To further improve the accuracy, a new vision transformer called Swin Transformer (Liu et al., 2021) was proposed, it is a hierarchical transformer whose representation is computed with Shifted windows. This hierarchical architecture has the flexibility of modeling at various scales and has linear computational complexity relative to the image size. These features make it compatible with many vision tasks, including image classification and semantic segmentation. Based on Swin Transformer, Cao et al. (2021) proposed a pure transformer U-shaped encoder-decoder network named Swin-Unet for medical image segmentation, which has relatively good performance in some datasets.

**Self-attention/transformer combined with CNN:** In recent years, researchers have tried to improve the performance of the network through the self-attention mechanism (Wang et al., 2018) to overcome the shortcomings of CNN learning global semantic information. In Schlemper et al. (2019), the skip-connections with additive attention gate were integrated with U-shaped architecture to improve medical image segmentation. But this is still the method based on CNN after all and it has not completely solved the limitation of learning global information. Several studies have been carried out to combine CNN and transformer. TransUNet (Chen et al., 2021) was proposed by combining the advantages of transformer and CNN. The transformer encodes image patches from a CNN feature map as the input sequence for extracting global contexts. A mixed transformer module (MTM) (Wang et al., 2021a) was proposed for simultaneous inter- and intra- affinities learning. TransFuse (Zhang et al., 2021) combines transformers and CNNs in a parallel style to capture both global dependency and low-level spatial details efficiently in a much shallower manner for medical image segmentations. Liang et al. (2022) proposed transconver with a parallel module named transformer-convolution inception which extracts local and global information *via* convolution blocks and transformer blocks, respectively. TransMed (Dai et al., 2021) was proposed for multi-modal medical image classification which combines the advantages of CNN and transformer to extract low-level features of images efficiently and establish long-range dependencies between modalities. These algorithms improve the global attention of the model based on their complementarity by directly combining CNN and transformer.

## 3. The Proposed Method

### 3.1. Overall Architecture Design

**Figure 1:**
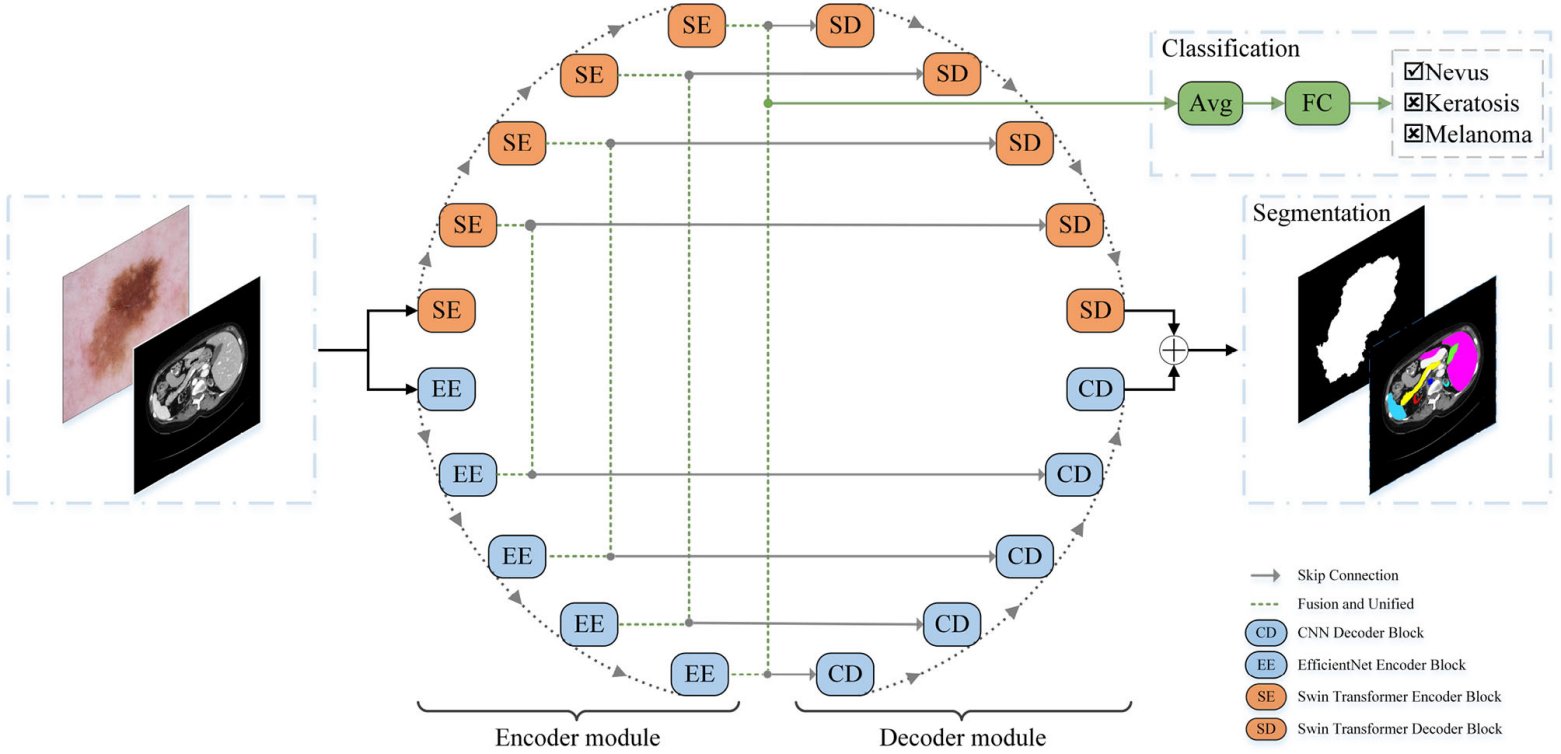
The architecture of our proposed O-Net.

A schematic view of the proposed O-Net is presented in Figure 1. O-Net is composed of two parts: an encoder module and a decoder module. The basic units of O-Net include the Swin Transformer block, EfficientNet block, and CNN Decoder block. During the segmentation task, the encoder module extracts the features of the input image to obtain the high-dimensional and low-dimensional features, which are then decoded back to the full spatial resolution by the decoder module. After extracting the features in the encoder part, the segmentation network provided an interface to integrate a classification network for simultaneously performing the classification task. Each module is described in detail below.

### 3.2. Swin Transformer Block

**Figure 2:**
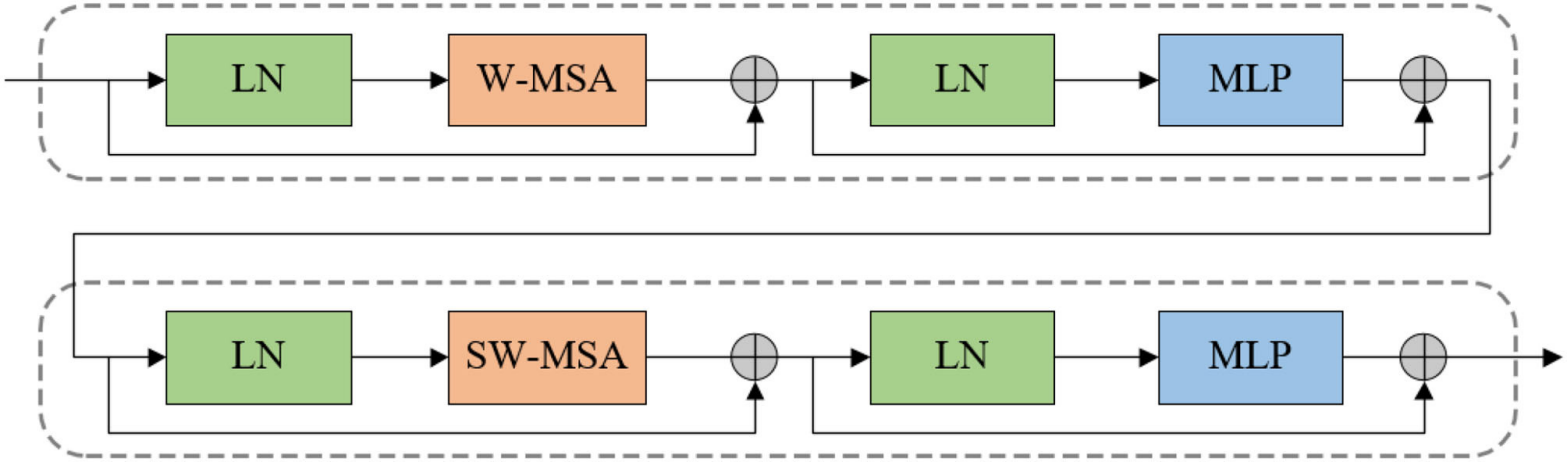
Two successive Swin Transformer block.

Different from the transformer, Swin Transformer is built based on shifted windows rather than the standard multi-head self attention (MSA) module. Two consecutive Swin Transformer blocks are presented in Figure 2. Each Swin Transformer block consists of residual connection and 2-layer MLP with Gaussian Error Linear Units (GELU) non-linearity, LayerNorm (LN) layer, and multi-head self attention module. The shifted window-based multi-head self attention (SW-MSA) module and the window-based multi-head self attention (W-MSA) module are applied in the two successive transformer blocks, respectively. Based on such a window partitioning approach, successive Swin Transformer blocks can be formulated as follows:


(1) $$\begin{array}{r} {ẑ}^{l}=W-MSA\left(LN\left(z^{l-1}\right)\right)+z^{l-1}, \end{array}$$

(2) $$\begin{array}{r} z^{l}=MLP\left(LN\left({ẑ}^{l}\right)\right)+{ẑ}^{l}, \end{array}$$

(3) $$\begin{array}{r} z^{l+1}=SW-MSA\left(LN\left(z^{l}\right)\right)+z^{l}, \end{array}$$

(4) $$\begin{array}{r} z^{l+1}=MLP\left(LN\left(z^{l+1}\right)\right)+z^{l+1}, \end{array}$$

Where *z*^*l*^ and ẑ^*l*^ represent the output features of the (S)W-MSA module and the MLP module of the *l*^*th*^ block, respectively. Similar to the previous works (Hu et al., 2018, 2019), self-attention is computed as follows:


(5) $$\begin{array}{r} Attention\left(Q,K,V\right)=SoftMax\left(\frac{QK^{T}}{\sqrt{d}}+B\right)V, \end{array}$$

where *Q, K, V*∈ℝ^*M*^^2^×*d* denote the query, key, and value matrices. M^2^ represents the number of patches in a window, and d is the query dimension. Since the relative position along each axis is within the range[−M+1, M−1], the values in B are taken from the bias matrix $\hat{B}\in {\mathbb{R}}^{\left(2M-1\right)\times 2M+1}$.

### 3.3. EfficientNet Block

**Figure 3:**
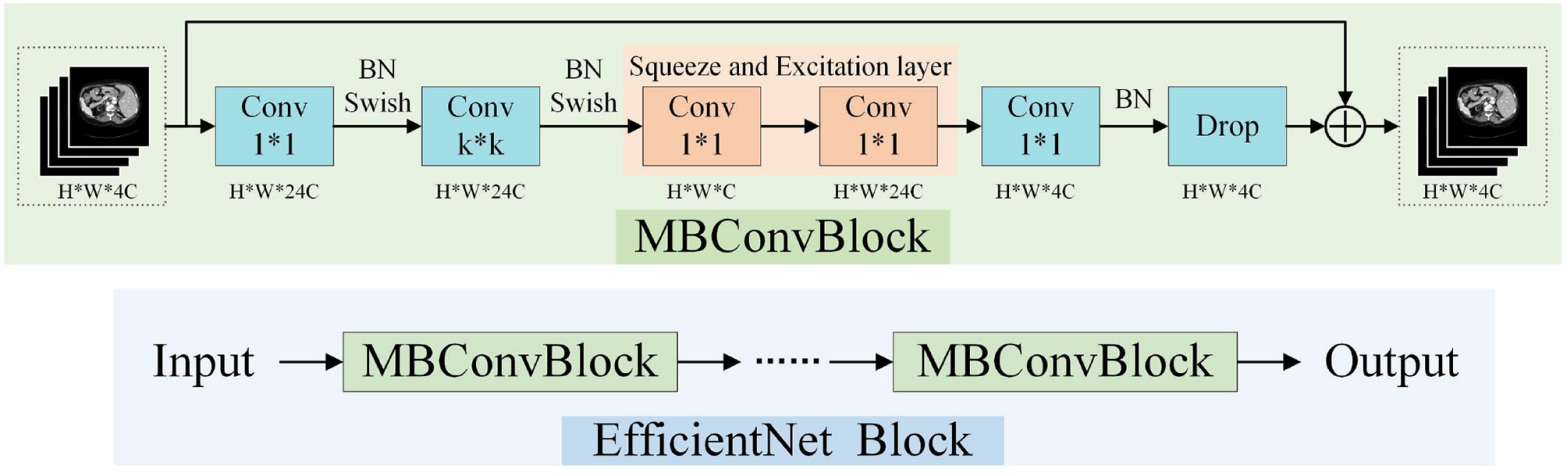
The architecture of EfficientNet Block.

EfficientNet block (Tan and Le, 2019) was proposed based on a neural structure search. This block uses composite coefficients to uniformly scale the depth, width, and resolution of the network. A schematic view of the EfficientNet block is presented in Figure 3. Each EfficientNet block is composed of MBConvBlocks (Sandler et al., 2018) which consists of convolution, batch normalization, and Swish activation layers. The network achieves better performance with the same parameters by uniformly scaling the network width, depth, or resolution in a fixed proportion. We employ the EfficientNet block as the encoder part of CNN to extract features efficiently and effectively.

### 3.4. Encoder Module

In the encoder part, we combine EfficientNet and Swin Transformer. For the Swin Transformer Encoder, it is composed of Swin Transformer Block and patch merging layer. Images are separated into non-overlapping patches with a patch size of 4 × 4 to transform the inputs into sequence embeddings, then concatenated together by the patch merging layer. The feature resolution will be down-sampled by 2 × after such processing, and the feature dimension of each patch becomes to 4 × 4 × 3 = 48. Furthermore, a linear embedding layer is applied to project feature dimension into an arbitrary dimension (represented as C). The transformed patch tokens pass through several Swin Transformer blocks and patch merging layers to generate the hierarchical feature representations.

For the EfficientNet encoder, the input image is convoluted and down-sampled first. Feature extraction is carried out through the EfficientNet block which uniformly scales the depth, width, and resolution of the network through composite coefficients. We can achieve relatively efficient feature extraction with only a small amount of computation using this module. Since the feature dimensions of two encoders are different, it is required to normalize the dimension before fusing them. The features extracted by the Swin Transformer are set to C × H × W using a linear projection. After that, the features are fused with the features extracted by the EfficientNet block *via* skip-connections. Similarly, when using the Swin Transformer decoder, we project the features extracted by the EfficientNet block through the linear embedding layer and fuse them with the features extracted by the Swin Transformer encoder.

### 3.5. Decoder Module

**Figure 4:**
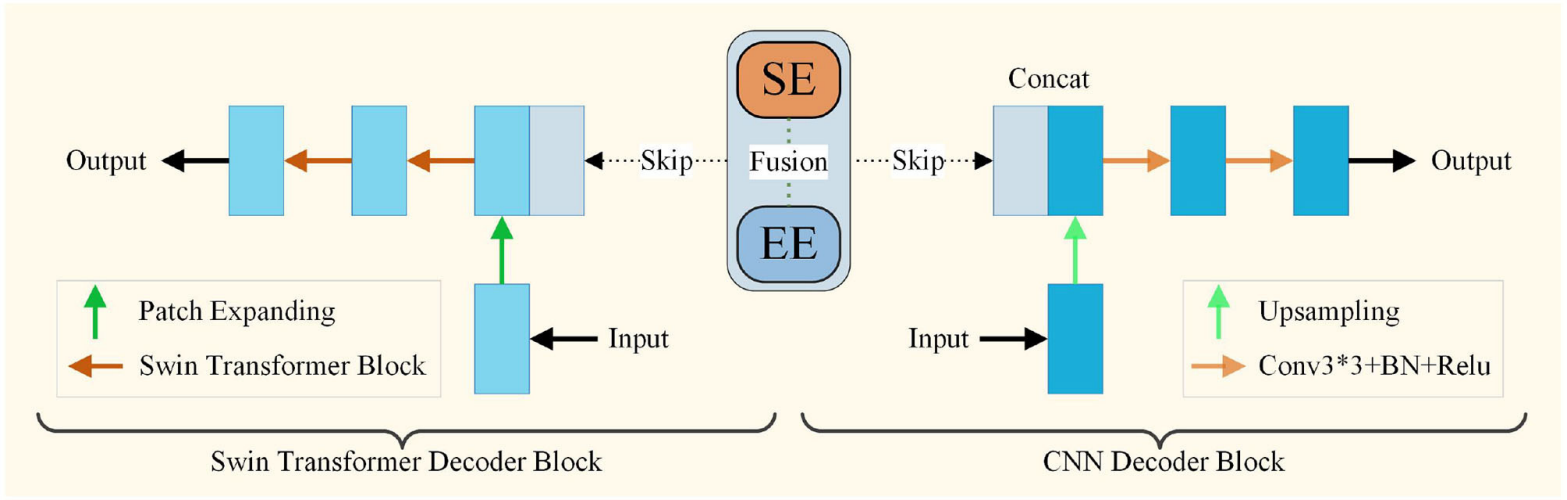
The architecture of decoder module.

The decoder module is adopted to restore the high-level semantic features extracted from the encoder module. The decoder part consists of the Swin Transformer decoder block and the CNN decoder block. A schematic view of the decoder modules is presented in Figure 4. The Swin decoder block is composed of a patch expanding layer and a Swin Transformer block. The features extracted by the encoder are multi-scale fused through skip-connections. The patch expanding layer reshapes feature maps of adjacent dimensions into large feature maps with 2 × up-sampling of resolution. In the end, the last patch expanding layer is used to perform 4 × up-sampling to restore the resolution of the feature maps to the input resolution (W × H), and a linear projection layer is applied on these up-sampled features to output the pixel-level segmentation predictions.

The CNN decoder block is composed of a 2 × upsampling operator, two 3 × 3 convolution layers, and a batch normalization layer with a Rectified Linear Units(ReLU) layer. Simple upsampling and convolution are two common operations of the decoder in the CNN decoder blocks. After the 2 × upsampling operator, the features were fused with those from encoders through skip-connection. After the two convolution processes, the features are input for the next decoder. At the end of the decoder, a convolution layer is applied to output the pixel-level segmentation predictions. Finally, the outputs of the two decoders are fused to obtain the final segmentation result.

### 3.6. Classification Method

The encoder part of the segmentation network and the classification network share the same structure. When encoders perform the classification task, the role of encoders is to extract contextual features and locate the target region like the segmentation task. The primary task of classification aims to accurately locate the target area, and the purpose of the segmentation network is to realize it. Therefore, after the training of the segmentation network, we use the learned weights of the encoder in the network as the initial parameters of the classification network. After that, we utilize the features of the lowest dimension in the encoder through the average pooling layer and a fully connected layer (FC) to perform the classification task.

## 4. Experiments

### 4.1. Datasets

**Synapse multi-organ segmentation dataset (synapse):** The dataset includes 30 abdominal CT scans from MICCAI 2015 Multi-Atlas Abdomen Labeling Challenge. Each CT volume consists of 85−198 slices of 512 × 512 pixels and there are 3,779 axial abdominal clinical CT images in total. Following Chen et al. (2021) and Liu et al. (2021), 18 samples were used as the training set and 12 samples as the testing set. The annotation of each image includes 8 abdominal organs (aorta, gallbladder, spleen, left kidney, right kidney, liver, pancreas, spleen, and stomach). The dice metric and the average Hausdorff Distance (HD) are used to evaluate our method on this dataset. The dice metric evaluates the degree of pixel overlap between the ground truth and prediction results and it is calculated as follows:


(6) $$\begin{array}{r} Dice=\frac{2\times TP}{2\times TP+FN+FP} \end{array}$$

where TP, FP, and FN refer to the number of true positives, false positives, and false negatives, respectively, besides, TN means true negatives. The HD calculates the maximum distance between the contours of the ground truth and predicted results, which can be formulated as follows:


(7) H(A,B) = max(h(A,B),h(B,A)

(8) $$\begin{array}{r} h\left(A,B\right)=\underset{a\in A}{\max}\left\{\underset{b\in B}{\min}\left\{∥a-b∥\right\}\right\} \end{array}$$

(9) $$\begin{array}{r} h\left(B,A\right)=\underset{b\in B}{\max}\left\{\underset{a\in A}{\min}\left\{∥b-a∥\right\}\right\} \end{array}$$

where A and B denote the contours of the ground truth and predicted results, respectively, and h(A,B) denotes the unidirectional HD from A to B.

**ISIC2017 skin lesion challenge dataset (ISIC2017):** The 2017 International Skin Imaging Collaboration (ISIC) skin lesion segmentation challenge dataset (Codella et al., 2018) includes 2,000 training images, 150 validation images, and 600 test dermoscopic images. Each image is paired with an expert manual tracing of skin lesion boundaries for the segmentation task and the lesion gold standard diagnosis (i.e., nevus, melanoma, and seborrheic keratosis) for the classification task. The size of the images in the dataset varies from 453 × 679 to 4499 × 6748 pixels. We used Dice, Mean Intersection over Union (IoU), Precision (Pre), Recall, F1-score, and Pixel Accuracy (PA) as the metrics to evaluate the accuracy of the segmentation work. In addition, we used Accuracy (AC), F1-score, precision (Pre), and specificity (SP) as the metrics to evaluate the classification task. These metric are calculated as follows:


(10) $$\begin{array}{r} IoU=\frac{TP}{TP+FN+FP} \end{array}$$

(11) $$\begin{array}{r} Pre=\frac{TP}{TP+FP} \end{array}$$

(12) $$\begin{array}{r} PA=\frac{TP+TN}{TP+TN+FP+FN} \end{array}$$

(13) $$\begin{array}{r} AC=\frac{TP+TF}{TP+TN+FP+FN} \end{array}$$

(14) $$\begin{array}{r} F1-score=\frac{2\times TP}{2\times TP+FP+FN} \end{array}$$

### 4.2. Implementation Details

Our method was implemented based on the Pytorch Deep Learning framework using python. For all training cases, flips and rotations were used as data augmentation to improve the generalization ability of the model. We trained our model on an Nvidia RTX 3090 GPU with 24GB memory. The input image size was set to 224 × 224 on the synapse dataset and 512 × 512 on the ISIC2017 dataset. The patch on the size was set to 4 in both tasks. All encoders and Swin Transformer blocks in the model were pretrained on ImageNet (Deng et al., 2009). During the training process of the synapse dataset, the batch size was set to 24 and the popular SGD optimizer with momentum of 0.9 and weight decay of 1e-4 and a learning rate of 1e-4 is used for the backpropagation of the model. During the process of ISIC2017 dataset, the models were optimized by AdamW with a learning rate of 1e-4 and a batch size of 8.

### 4.3. Experiment Results on the Synapse Dataset

**Figure 5:**
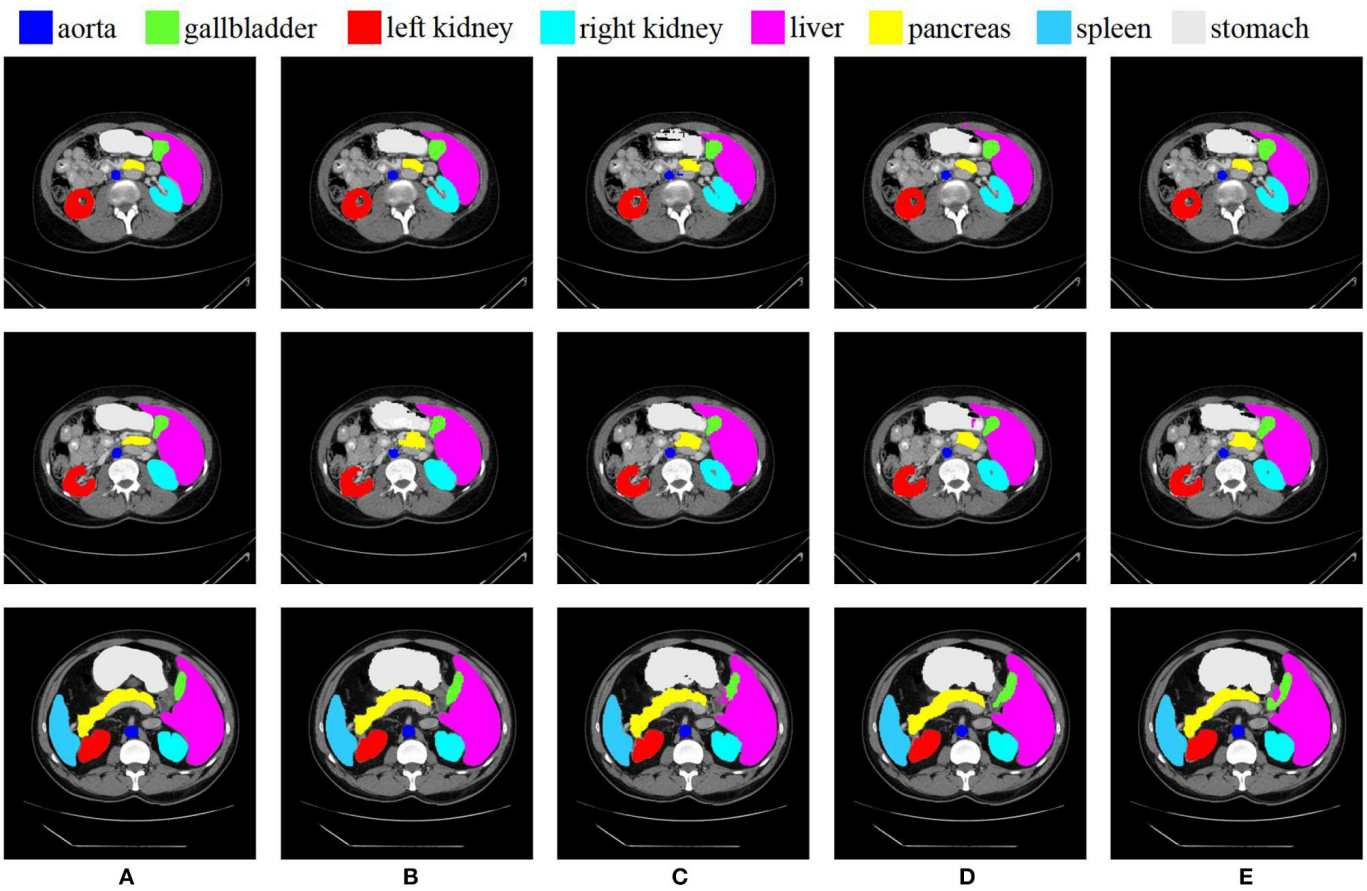
Conparision of different methods on the Synapse multi-organ dataset by visualization. From left to right: **(A)** Ground Truth, **(B)** O-Net, **(C)** SwinUNet, **(D)** TransUNet, and **(E)** R50 AttUNet.

The comparison of the proposed O-Net with previous state-of-the-art methods on the synapse multi-organ CT dataset is presented in Table 1. Experimental results demonstrate that our algorithm achieves the best performance with a segmentation accuracy of 80.61% (Dice↑) and 21.04 (HD↓) performance. We can see from the results that the CNN-based method performs worse on edge predictions than the transformer method from the metric of HD. This also indicates that our algorithm not only performs better in terms of segmentation, but also has a good performance in edge prediction. For organs with high segmentation difficulty such as Pancreas and Gallbladder, our method obtains the best and the third results, respectively, which also reflects the strong generalization of our algorithm. The specific segmentation results of different algorithms on this dataset are presented in Figure 5. In this work, we demonstrate that the in-depth combination of CNN and Swin Transformer can learn both the global and the local contextual features, thereby obtaining better segmentation results.

**Table 1:** Experimental results of different methods on the synapse multi-organ CT dataset.

**Method**	**Dice↑**	**HD↓**	**Aorta**	**Gallbladder**	**Kidney(L)**	**Kidney(R)**	**Liver**	**Pancreas**	**Spleen**	**Stomach**
V-Net Milletari et al. (2016)	68.81	–	75.34	51.87	77.10	**80.75**	87.84	40.05	80.56	56.98
DARR Fu et al. (2020)	69.77	–	74.74	53.77	72.31	73.24	94.08	54.18	89.90	45.96
R50 ViT Chen et al. (2021)	71.29	32.87	73.73	55.13	75.80	72.20	91.51	45.99	81.99	73.95
U-SegNet Kumar et al. (2018)	72.61	43.94	85.69	64.33	75.12	66.41	91.72	50.59	84.07	62.96
R50 U-Net Chen et al. (2021)	74.68	36.87	87.74	63.66	80.60	78.19	93.74	56.90	85.87	74.16
AION Gehlot and Gupta (2021)	75.54	32.27	87.59	58.74	82.47	73.45	93.47	49.44	87.52	71.61
R50 Att-UNet Chen et al. (2021)	75.57	36.97	55.92	63.91	79.20	72.71	93.56	49.37	87.19	74.95
U-Net Ronneberger et al. (2015)	76.85	39.7	89.07	**69.72**	77.77	68.60	93.43	53.98	86.67	75.58
EDNFC-Net Gehlot et al. (2020)	77.21	35.07	86.08	62.47	84.31	78.27	92.61	57.31	85.36	71.24
TransUNet Chen et al. (2021)	77.48	31.69	87.23	63.13	81.87	77.02	94.08	55.86	85.08	75.62
Att-UNet Oktay et al. (2018)	77.77	36.02	**89.55**	68.88	77.98	71.11	93.57	58.04	87.30	75.75
TransFuse Zhang et al. (2021)	78.95	26.59	87.09	61.64	82.20	76.91	94.19	59.01	89.86	80.73
Swin-Unet Cao et al. (2021)	79.13	21.55	85.47	66.53	83.28	79.61	94.29	56.58	**90.66**	76.60
O-Net	**80.61**	**21.04**	88.36	67.45	**84.44**	77.13	**95.24**	**61.52**	90.03	**80.74**

*The symbol ↑ means the higher value, the better*.

*The symbol ↓ means the lower value, the better.*

*Bold font to highlight the optimal values*.

### 4.4. Experiment Results on the ISIC2017 Dataset

**Figure 6:**
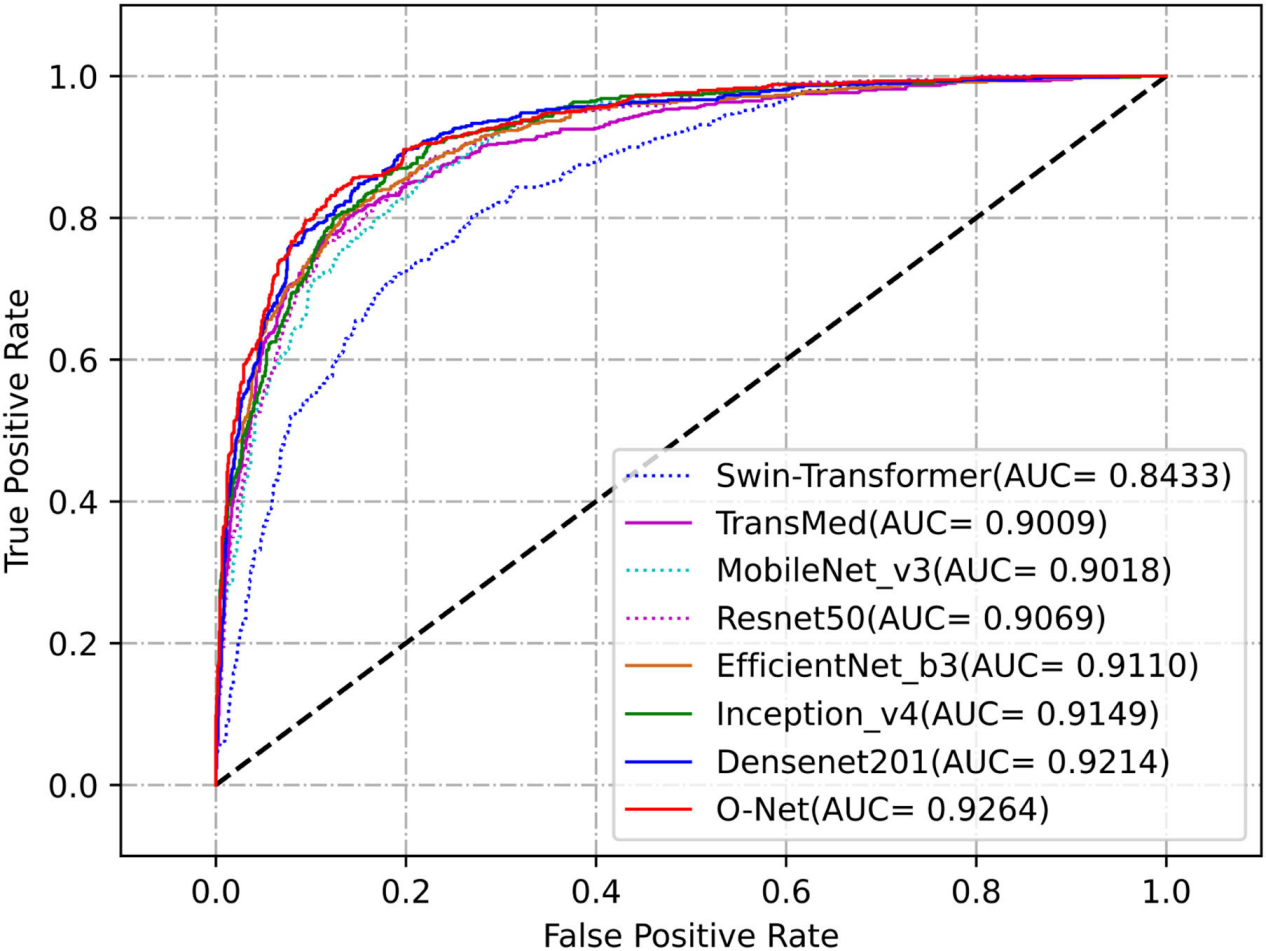
Receiver Operating Characteristic curves of the different methods for classification task on the ISIC2017 dataset.

**Figure 7:**
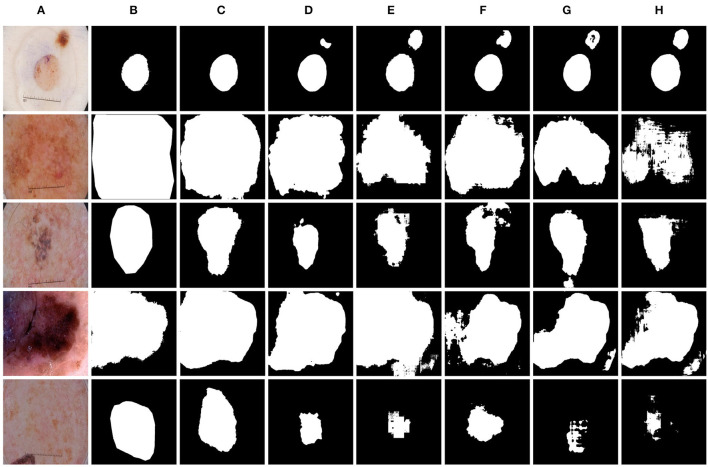
Comparison of different methods on the ISIC2017 dataset by visualization. **(A)** Image, **(B)** Ground Truth, **(C)** O-Net, **(D)** TransUNet, **(E)** Swin-UNet, **(F)** CE-Net, **(G)** R50 AttUNet, and **(H)** UNet.

We further evaluated the proposed method for medical image segmentation and classification using the ISIC2017 dataset. The results of segmentation and classification are presented in Tables 2, 3. From Table 2, we can see that in the segmentation task, the combination of the CNN and the Swin Transformer can achieve better performance than that of single CNN or that of only the Swin Transformer. This indicates the effectiveness of the combination of these two structures. The O-Net has achieved the best performance in the six metrics which reflects the superiority of our method. The Receiver Operating Characteristic (ROC) curves of the classification methods are shown in Figure 6. The Area Under Curve (AUC) value for O-Net is 0.9264 which is the best performance among compared methods. Based on the data from Table 3 and the ROC curves of the classification task, we can see that O-Net has also achieved excellent performance in the classification task. The specific segmentation results of different algorithms on this dataset are presented in Figure 7. The experimental results of classification tasks on this dataset indicate that combining CNN and Swin Transformer for classification tasks can improve the accuracy of the classification tasks. The performance can be further improved by initializing the classification network with the parameters from the encoder part of the segmentation network.

**Table 2:** Segmentation results of different methods on the ISIC2017 dataset.

**Method**	**Dice**	**mIoU**	**Pre**	**recall**	**F1-score**	**PA**
U-Net Ronneberger et al. (2015)	85.22	78.40	91.17	73.98	77.80	91.19
R50-U-Net Xiao et al. (2018)	87.48	80.86	92.99	78.19	81.70	92.19
U-SegNet Kumar et al. (2018)	87.87	81.22	90.50	81.13	82.49	92.33
ENDFC-Net Gehlot et al. (2020)	88.00	81.43	90.26	**82.10**	82.80	92.29
M-Net Fu et al. (2018)	88.33	82.25	94.46	79.04	83.38	92.67
AION Gehlot and Gupta (2021)	88.84	82.56	92.26	81.95	84.02	92.88
CE-Net Gu et al. (2019)	89.64	83.56	95.40	80.47	84.99	93.67
Swin-Unet Cao et al. (2021)	88.77	82.69	94.64	79.16	83.51	94.04
TransFuse Zhang et al. (2021)	89.63	83.78	95.56	80.35	84.75	93.73
TransUNet Chen et al. (2021)	89.99	84.21	95.59	81.21	85.42	93.97
**O-Net**	**90.30**	**84.52**	**95.65**	81.72	**85.89**	**94.09**

*Bold font to highlight the optimal values*.

**Table 3:** Classification accuracy of different methods on the ISIC2017 dataset.

	**Average**	**Nevus classification**				
**Method**	**AC**	**AC**	**F1-score**	**Pre**	**SP**				
Swin Transformer Liu et al. (2021)	80.22	89.50	81.18	62.16	91.76				
AION Gehlot and Gupta (2021)	81.55	85.33	76.01	50.74	86.86				
TransMed Dai et al. (2021)	84.11	89.19	80.10	61.90	92.16				
MobileNetV3 Howard et al. (2019)	84.89	89.33	81.53	60.83	90.78				
EfficientNet-B3 Tan and Le (2019)	85.22	90.67	82.64	66.67	93.33				
Inception v4 Szegedy et al. (2016)	85.33	89.16	81.45	60.16	90.39				
ResNet50 He et al. (2016)	85.44	91.00	82.97	68.37	93.92				
DenseNet201 Huang et al. (2017)	86.56	**92.00**	**85.36**	69.81	93.73				
O-Net	**87.22**	91.67	83.51	**72.73**	**95.29**				
	**Average**	**Melanoma classification**				**Keratosis classification**			
**Method**	**AC**	**AC**	**F1-score**	**Pre**	**SP**	**AC**	**F1-score**	**Pre**	**SP**
Swin Transformer Liu et al. (2021)	80.22	73.00	71.63	84.07	73.91	78.17	68.45	45.33	83.02
AION Gehlot and Gupta (2021)	81.55	77.33	75.69	85.40	74.40	82.00	69.73	54.46	90.48
TransMed Dai et al. (2021)	84.11	79.17	77.13	84.72	71.50	84.00	79.83	59.63	55.56
MobileNetV3 Howard et al. (2019)	84.89	80.50	78.78	86.51	75.36	84.83	74.59	62.75	92.13
EfficientNet-B3 Tan and Le (2019)	85.22	80.50	78.82	86.70	75.85	84.50	**75.71**	59.84	89.86
Inception v4 Szegedy et al. (2016)	85.33	80.83	79.05	86.39	74.88	86.00	75.94	67.37	93.58
ResNet50 He et al. (2016)	85.44	81.50	79.99	**87.90**	**78.26**	83.83	75.28	57.69	88.61
DenseNet201 Huang et al. (2017)	86.56	83.50	81.55	86.57	73.91	84.17	72.48	61.96	92.75
O-Net	**87.22**	**84.17**	**81.58**	84.49	67.63	**85.83**	74.19	**70.00**	**95.03**

*Bold font to highlight the optimal values*.

### 4.5. Ablation Study

The results of the ablation studies are shown in Tables 4, 5. We will compare and analyze the effects of different factors on the segmentation performance in the following sections.

**Table 4:** Ablation study on the encoder of CNN method.

**Encoder**	**Params**	**Dice↑**	**HD↓**	**Aorta**	**Gallbladder**	**Kidney(L)**	**Kidney(R)**	**Liver**	**Pancreas**	**Spleen**	**Stomach**
MobileNetV3 Howard et al. (2019)	**5.48**	76.66	26.27	86.12	62.25	82.07	**90.65**	94.06	55.72	88.62	73.77
DenseNet201 Huang et al. (2017)	20.01	78.91	20.45	87.52	65.52	82.61	78.20	95.05	57.42	86.40	78.65
Resnet50 He et al. (2016)	25.55	79.16	23.01	87.71	66.86	81.73	75.22	94.18	58.86	**90.42**	78.31
Inception v3 Szegedy et al. (2016)	23.83	80.36	22.78	88.09	63.76	82.19	79.25	95.16	**65.17**	87.12	**82.13**
EfficientNet-b3 Tan and Le (2019)	12.23	**80.61**	**21.04**	**88.36**	**67.45**	**84.44**	77.13	**95.24**	61.52	90.03	80.74

*The symbol ↑ means the higher value, the better*.

*The symbol ↓ means the lower value, the better.*

*Bold font to highlight the optimal values*.

**Table 5:** Ablation study on the combination of CNN method and Swin Transformer method.

**Encoder**		**Decoder**			
**Efficient**	**Swin**	**CNN**	**Swin transformer**	**Dice↑**	**HD↓**	**Aorta**	**Gallbladder**	**Kidney(L)**	**Kidney(R)**	**Liver**	**Pancreas**	**Spleen**	**Stomach**
**net-block**	**transformer**	**decoder**	**decoder**	
✓		✓		78.86	28.86	87.72	62.19	83.11	76.67	94.49	56.61	89.48	80.58
✓			✓	79.93	26.88	87.90	68.09	83.89	76.05	94.42	**62.95**	87.32	78.86
✓		✓	✓	80.34	22.53	**88.67**	67.38	83.95	77.01	95.12	60.06	88.76	**81.77**
	✓	✓		77.55	31.03	86.14	63.49	81.59	75.82	93.68	54.61	90.19	74.87
	✓		✓	79.13	21.55	85.47	66.53	83.28	79.61	94.29	56.58	**90.66**	76.60
	✓	✓	✓	79.38	22.34	87.60	62.53	84.86	**80.54**	94.42	58.75	90.64	75.66
✓	✓	✓		79.47	29.19	87.71	66.21	81.64	74.69	94.65	61.61	89.19	80.02
✓	✓		✓	80.41	27.33	86.74	71.19	84.32	77.29	94.30	60.63	89.2	79.64
✓	✓	✓	✓	**80.61**	**21.04**	88.36	**67.45**	**84.44**	77.13	**95.24**	61.52	90.03	80.74

*The symbol ↑ means the higher value, the better*.

*The symbol ↓ means the lower value, the better*.

*Bold font to highlight the optimal values*.

**Effect of encoder:** The experimental results in Table 4 show that the best results are achieved by using the EfficientNet block as the encoder, while the number of parameters is not large. The parameter quantity of the MobileNet is smaller than that of the EfficientNet, but its accuracy is far too poor than the others. The accuracy of Inception v3 is similar to ours, but the amount of calculation is much larger than that of EfficientNet. Therefore, we use EfficientNet as a CNN-based encoder.

**Effect of combination:** The segmentation network consists of encoder and decoder. How to combine the CNN based method and the Swin Transformer based method is a point worth exploring. Table 5 shows the effects of adopting different models for encoder and decoder. It can be seen from the results that the best performance is achieved by combining them in both the encoder and decoder parts. As can be seen from the results, better segmentation performance is achieved when CNN is used in the encoder part and Swin Transformer is used in the decoder part.

**Effect of learning rate and batch size:** To explore the best learning rate and batch size in the training process of the algorithm, we carried out a series of experiments. The experimental results are shown in Table 6. It can be seen from the top half of the chart that the best Dice was obtained when the learning rate was set to 1e-2. Although the best HD was obtained when the learning rate was set to 1e-1, its Dice was lower, therefore, we chose the learning rate of 5e-2. We can also draw from the bottom half of the chart that the best dice was obtained when the batch size was set to 24. Although the HD is lower when batch size was set to 8 and 6, the Dice of the Gallbladder is far too low, which is not conducive to the overall segmentation, therefore, the batch size of 24 would be more appropriate.

**Table 6:** Ablation study on learning rate and batch size.

**Learn rate**	**Dice↑**	**HD↓**	**Aorta**	**Gallbladder**	**Kidney(L)**	**Kidney(R)**	**Liver**	**Pancreas**	**Spleen**	**Stomach**
1e-1	79.21	**20.06**	86.56	63.48	**84.61**	**77.14**	94.32	56.99	**91.90**	78.69
5e-2	**80.61**	21.04	**88.36**	67.45	84.44	77.13	**95.24**	**61.52**	90.03	80.74
1e-2	79.07	20.14	87.64	67.74	81.95	74.69	94.71	58.33	89.44	78.03
5e-3	79.76	23.07	88.18	**68.51**	83.60	76.92	94.42	58.84	88.59	79.03
1e-3	76.57	30.37	85.28	62.96	81.61	74.51	92.96	54.13	86.37	**84.70**
**Batch size**	**Dice**↑	**HD**↓	**Aorta**	**Gallbladder**	**Kidney(L)**	**Kidney(R)**	**Liver**	**Pancreas**	**Spleen**	**Stomach**
8	78.81	**15.67**	88.12	44.45	**84.59**	**80.24**	94.73	**67.40**	89.97	81.00
16	78.36	18.25	**88.69**	38.12	84.57	79.46	95.16	66.20	**91.44**	**83.27**
24	**80.61**	21.04	88.36	**67.45**	84.44	77.13	95.24	61.52	90.03	80.74
32	80.35	27.93	88.32	66.70	81.94	76.19	**95.31**	64.06	88.94	81.27

*The symbol ↑ means the higher value, the better*.

*The symbol ↓ means the lower value, the better.*

*Bold font to highlight the optimal values*.

## 5. Conclusion

We introduce a novel method based on the combination of CNN and Swin Transformer for medical image segmentation and classification. To make full use of the global and the local information to improve medical image segmentation and classification, we propose O-Net, which combines the advantages of these two structures for improving both the segmentation and the classification performance. We combine CNN and transformer in both encoder and decoder parts of the network. In addition, we have shown that the proposed segmentation network is beneficial for the classification task. Experimental results have demonstrated that the proposed O-Net achieves competitive performance and good generalization ability in both the segmentation and the classification tasks.

## Data Availability Statement

The original contributions presented in the study are included in the article/supplementary material, further inquiries can be directed to the corresponding authors.

## Author Contributions

TW, JL, ZHa, ZHu, YH, YD, QG, MD, TT, and GC: concept and design. TW, JL, HZ, JW, MC, and TT: acquisition of data. TW, JL, ZHa, ZHu, QG, and TT: model design. TW, JL, ZHa, ZHu, YH, YD, and TT: data analysis. TW, JL, ZHa, ZHu, YH, YD, TT, and GC: manuscript drafting. TW, JL, ZHa, ZHu, YH, YD, HZ, JW, MC, HJ, R-GL, QG, MD, TT, and GC: approval. All authors contributed to the article and approved the submitted version.

## Funding

This work was supported in part by the National Natural Science Foundation of China under Grant Nos. 61901120 and 62171133, the Science and Technology Program of Fujian Province of China under Grant No. 2019YZ016006, and Health and Family Planning Research Talent Training Program of Fujian Province under Grant No. 2020GGB009.

## Conflict of Interest

QG and TT were employed by Imperial Vision Technology. The remaining authors declare that the research was conducted in the absence of any commercial or financial relationships that could be construed as a potential conflict of interest.

## Publisher's Note

All claims expressed in this article are solely those of the authors and do not necessarily represent those of their affiliated organizations, or those of the publisher, the editors and the reviewers. Any product that may be evaluated in this article, or claim that may be made by its manufacturer, is not guaranteed or endorsed by the publisher.
